# CDK/CCN and CDKI Alterations for Cancer Prognosis and Therapeutic Predictivity

**DOI:** 10.1155/2014/361020

**Published:** 2014-01-29

**Authors:** Patrizia Bonelli, Franca Maria Tuccillo, Antonella Borrelli, Antonietta Schiattarella, Franco Maria Buonaguro

**Affiliations:** Molecular Biology and Viral Oncology Unit, Department of Research, Istituto Nazionale Tumori-IRCCS Fondazione “G. Pascale”, 80131 Naples, Italy

## Abstract

The regulation of cell growth and division occurs in an accurate sequential manner. It is dictated by the accumulation of cyclins (CCNs) and cyclin-dependent kinases (CDKs) complexes and degradation of CCNs. In human tumors, instead, the cell cycle is deregulated, causing absence of differentiation and aberrant cell growth. Oncogenic alterations of CCNs, CDKs, and CDKIs have been reported in more than 90% of human cancers, and the most frequent are those related to the G1 phase. Several molecular mechanisms, including gene overexpression, chromosomal translocations, point mutations, insertions and deletions, missense and frame shift mutation, splicing, or methylation, may be responsible for these alterations. The cell cycle regulators are involved in tumor progression given their association with cancers characterized by higher incidence of relapses and chemotherapy resistance. In the last decade anticancer drug researches focused on new compounds, able to target molecules related to changes in genes associated with tumor status. Recently, the studies have focused on the restoration of cell cycle control modulating molecular targets involved in cancer-cell alterations. This paper aims to correlate alterations of cell cycle regulators with human cancers and therapeutic responsivity.

## 1. Introduction

The recent progress in the field of molecular medicine has identified several molecular markers involved in the regulation of the cell cycle as a target for prognosis and cancer treatment. Cell cycle is deregulated in human tumors, causing the absence of differentiation and aberrant cell growth [1–3]. The cell cycle includes cell division, differentiation, growth, and programmed cell death through apoptosis. The regulation of this process involves environmental stimuli that lead to the activation of cyclin-dependent serine/threonine kinases (CDKs), regulated by cyclins (CCNs) and inhibitors of cyclin-dependent kinases (CDKIs). The main phases regulated by CDKs are the DNA integrity control checkpoints, mediated by the retinoblastoma susceptibility gene suppressor (*Rb*), the tumor suppressor gene *TP53*, and transcription factors of the E2F family [4, 5].

So far nine CDKs and at least 15 CCNs have been identified [6–8]. The CDKs, proteins of 300 amino acids in length, are activated by a no covalent binding with specific cyclins triggering the transition between different phases of the cell cycle. The formation of the complex CCN/CDK is usually transient and is regulated by ubiquitin-mediated degradation. The CDKs are negatively regulated by endogenous inhibitors CDKIs. So far two families of inhibitors have been identified: the p21 and the p16 families. The p21 family includes p21/CDKN1A, p27/CDKN1B, and p57/CDKN1C [9, 10]. The p16 family includes p16/CDKN2A, p15/CDKN2B, p18/CDKN2C, and p19/CDKN2D [9]. The p21 family members interact with both CCNs and CDKs subunit, while members of the p16 family interact only with the CDKs [7].

## 2. Cell Cycle Regulators

**Figure 1:**
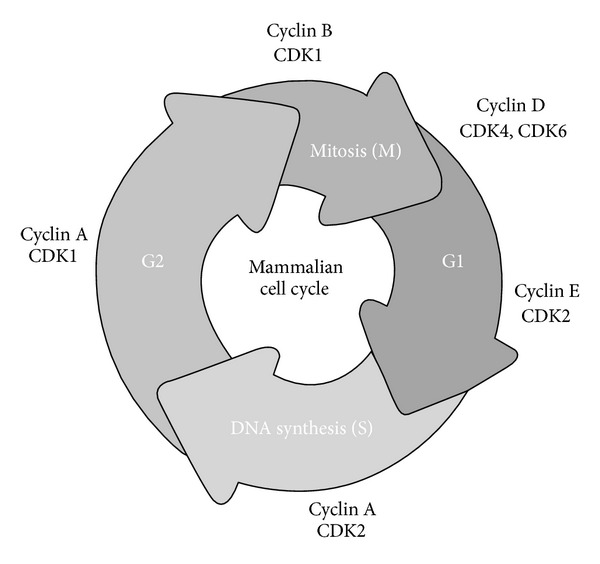
The mammalian cell cycle. Cyclins and cyclin-dependent kinases are referred to their specific phase of the cell cycle. Following mitogenic signals that promote the cell entry in the G1 phase, the progression through the cell cycle is regulated by sequential activation of cyclins and cyclin-dependent kinases.

In Figure 1, an overview of the mammalian cell cycle is reported. The regulation of cell growth and division occurs in a precise sequential manner. It is dictated by the accumulation of CDK/CCN complexes and degradation of CCNs. Quiescent cells in G0 enter the G1 phase in response to external stimuli, such as mitogenic growth factors, or on the basis of internal needs. Cyclin D (CCND) binds to and activates CDK4 and/or CDK6, depending on the cell type. The complex CCND/CDK4 or CDK6 phosphorylates Rb. The cyclin E (CCNE) binds to and activates CDK2, resulting in phosphorylation of a different site on Rb. The E2F transcription factors (EF1–EF5) dissociate from the hyperphosphorylated form of Rb to activate the transcription of genes that promote S phase including the thymidylate synthase (TS), the dihydrofolate reductase (DHFR), and the DNA polymerase (POL) [11, 12]. From this point on, the cell has passed the “restriction point” and becomes committed to the progression in the cell cycle and independent of growth factors. CCNA and CCNE bind to CDK2 allowing the cell to proceed through the S phase. The complex CCNA/CDK2 facilitates the transition from the S phase to the G2 phase. The complexes CCNB/CDK1 accumulate in late G2 phase, necessary for the progression of the cell through the M phase [13]. Following completion of anaphase, CCNB is degraded, thus returning the cell to G1 state, which, in the presence of stimulation by growth factors, proceeds by successive cycles of cell division. CDC25 (A, B, and C) are also required for the activation of CDK complexes that control progression through the cell cycle. Activation of CDKs can be achieved through dephosphorylation by members of the CDC25 phosphatase family (CDC25A, CDC25B, and CDC25C). CDC25A plays an important role at the G1/S-phase transition. CDC25B undergoes activation during S phase and plays a role in activating the mitotic kinase CDK1/CCNB in the cytoplasm. Active CDK1/CCNB then phosphorylates and activates CDC25C, leading to a positive feedback mechanism and to entry into mitosis [14]. The repair of DNA damage or apoptosis occurs prevalently at checkpoint G1, while the integrity of the synthesized DNA is examined at G2, to ensure the fidelity of the replicated genome [15, 16]. The CDKs play an important role in the regulation of these checkpoints. For example, in response to various stress signals, TP53, a transcription factor, is activated and causes the transcriptional induction of CDKN1A and the cell cycle arrest at the G1 checkpoint [10]. The length of the individual phases of the cell cycle can vary depending on the cell type and the particular conditions. The activity of CDKs during the cell cycle is controlled at multiple levels including the association with CCN, activating transient expression and rapid degradation of CCNs, posttranslational modifications by kinases and phosphatases, interactions with CDKIs, and intracellular translocations [9].

## 3. Cell Cycle in Cancer

Oncogenic alterations of CCNs, CDKs, CDKIs, and other components of Rb pathway have been reported in more than 90% of human cancers [1, 2, 17–40] as summarized in Table 1.

One of the most relevant alterations is represented by the p16 (CDKN2A)-CCNDs/CDK-Rb pathway frequently altered in various types of cancers [41–43]. In solid tumors, there is a correlation between genetic alterations within this pathway and clinical outcome in cancer patients [44]. Anomalies of CDKs and CCNDs are very frequent in the G1 phase. Several molecular mechanisms may be responsible for these alterations, including gene overexpression, chromosomal translocations, point mutations, insertions and deletions, missense and frame shift mutation, splicing, or methylation. CDKs are found overexpressed in several types of tumors, including sarcoma, colon carcinoma, and lymphoma [45–47]. CDK overexpression can be caused by gene amplification [48], chromosome translocation, or point mutations [49] that impair the kinase interaction with CDK inhibitors, as the case for CDK4 in some melanoma patients [50]. CDK activity can also be dysregulated by overexpression of the cyclin partner or inactivation of CDK inhibitors, both events being quite common in tumors [51, 52]. The lack of regulation of CDKs by its inappropriate activation is essential for maintaining the malignant transformation. Changes of *CCND1* gene expression have been reported in several neoplasias. In particular, *CCND1* gene is induced (transactivation) by various oncogenic signals including the activating mutation of ras genes, src, and mitogen-activated protein kinases (MAPK) [53, 54], as well as myc [55, 56]. Moreover, chromosomal aberrations involving CCND1 have been reported in B-lymphocytic malignancy and multiple myeloma [57, 58]. CCND1 overexpression played a role in the pathogenesis of mammary cancer in transgenic mice [59, 60] and lymphoma [61]. The dysregulation of CCNE is associated with hyperproliferation and malignant transformation [26]. Overexpression of CCNE1 has been linked to endometrial hyperplasia and/or carcinoma [25]. CCNE1 is overexpressed in many human tumors, in particular, breast cancer, and also nonsmall cell lung cancer, leukemia, and others [62]. CCNE has been found to be amplified, overexpressed, or both in some cases of breast and colon cancer and in acute lymphoblastic and myeloid leukaemia [63–65].

## 4. Clinical Implication of Cell Cycle Dysregulation

### 4.1. Cell Cycle and Cancer Prognosis

The cell cycle regulators, as CCNs and CDKIs, are involved in the mechanisms of tumor progression. CCND is associated with higher incidence of relapses in tumors of the head and neck [66] and in chemotherapy resistance [67]. Tumors that overexpress CCND1 generally have a poor prognosis [68–70]. Also overexpression of CCNE has been reported to be a poor prognostic factor in cancers of various organs [71–73]. Transgenic mice overexpressing human CCNE spontaneously developed mammary carcinoma [74]. CCNE overexpression correlates well with the aggressiveness of breast cancer [75], with gastric cancer progression [76], and is predictive of the risk of distant recurrence in the abdomen [77]. The inactivation of endogenous inhibitors of p16 or p21 family, due to their mutation/deletion or TP53-mediated changes, causes aberrant activity of CDK and inactivation of Rb. The loss of *CDKN2A, CDKN1B,* and* CDKN1A *is a predictor of poor prognosis in several types of cancer [78–83]. The loss of CDKN2A appears to be closely related to the functional inactivation of CDKN1B, and assessment of CDKN1A status may be useful for a precise prognostic prediction of individuals with HCC expressing high levels of CDKN1B [84]. Hypermethylation of *CDKN1A *promoter suppresses *CDKN2A* expression with a subsequent poor prognosis in patients with esophageal squamous cell carcinomas [85]. Loss of *CDKN1B* was associated with poor prognosis in patients with Dukes' B tumor or those with proximal tumor [80] and in patients with pancreatic cancer [81]. Tenjo et al. [82] observed that altered *CDKN1B* expression was a predictor of poor prognosis for patients with stage III colorectal cancers. Codeletion of *CDKN2B/CDKN2A* genes is significantly related to the prognosis of NSCLC patients, whereby detecting codeletion of both genes might be used as a potential marker for NSCLC prognosis [83]. The *CDKN2A/CDKN2B *deletion correlates with a high risk of relapse or death in patients with ALL [86, 87]. Myelodysplastic syndromes patients with *CDKN2B* gene methylation at diagnosis or in subsequent studies had a significantly higher chance of disease progression to AML than those without the gene methylation [88]. The CDKN1B protein negatively regulates G1 progression by binding to G1 CCN/CDK complexes and inhibits their activity, resulting in inhibition of entry to the cell cycle. Reduced levels of CDKN1B occur in several cancer types and are generally associated with poor prognoses. For example, loss of *CDKN1B* has been revealed to be an independent prognostic factor in breast, colon, and gastric carcinomas [89, 90]. Gastric tumors with high CDKN1B were well differentiated, with low levels of invasion and lymph node metastasis. CDKN1B-negative cases demonstrated a poor prognosis [91]. Expression of *CDKN1B* is significantly decreased in renal cell carcinoma (RCC) as compared with normal kidney tissue. Loss of *CDKN1B* expression is a risk factor for disease recurrence and the strongest predictor of cancer-specific survival [92].

The expression of *CDKN1A* gene acts as an inhibitor of the cell cycle during G1 phase and is tightly controlled by the tumor suppressor protein TP53. Normal cells generally display a rather intense nuclear *CDKN1A* expression. Loss of *CDKN1A* expression has been associated with poor prognosis in several carcinomas [93].

Recently, it has been demonstrated that microRNAs (miRNAs), a class of small noncoding RNAs, control the regulators of cell cycle, modulating their gene expression. miR-24 directly targets CDKN1B and CDKN2A in keratinocytes and in different cancer-cell lines promoting their proliferation. It is involved in posttranscriptional regulation of CDKIs, and its upregulation may play a role in carcinogenesis [94]. Several studies showed that CDKN1A could be regulated at the translational level by miRNA such as miR-93, miR-20a/b, miR-17, and miR-106a/b [95, 96]. Wu et al. have demonstrated that CDNK1A can be directly targeted and modulated by multiple miRNA molecules [97].

### 4.2. Cell Cycle and Therapeutics

Although chemotherapeutic drugs save many lives, they are very toxic, nonselective, and less effective than desired. In the last decade anticancer drug researches focused on new compounds, able to target molecules related to changes in genes associated with tumor status. Recently, the studies have focused on the restoration of cell cycle control modulating molecular targets involved in cancer-cell alterations. Several potential strategies have been proposed such as inhibition of CDKs, downregulation of cyclins, overexpression of endogenous CDKIs, disruption of the interactions CCN/CDK, altered proteolysis, degradation of CCNs, and specific inhibition of tyrosine kinase leading to activation of the cell cycle. In Table 2, several compounds have been reported according to their molecular targets. The response of the tumors to modulators of cell cycle varies from simple cytostasis to cell death depending on the concentrations used and the downstream result of the cell cycle arrest. 7-Hydroxystaurosporine (UCN-01) has shown antitumor activity against several human cancer-cell lines. UCN-01 inhibits the cell cycle progression from the G1 to the S phase and is associated with inhibition of cyclin-dependent kinase (CDK) activity and induction of CDKN1A. The combination of UCN-01 and tamoxifen results in augmented cytotoxicity and may have a potential clinical application in the treatment of breast cancer [98]. Butyrolactone 1, inhibitor of CDKI, accelerates CCNB1 accumulation in G2/M of renal cells which shifted to G1 phase without cell division [99]. Indirubin-3′-oxime (I3M), an indigo alkaloid, was found to display potent antitumor activities on various types of cancer cells. I3M increased the level of CDKN1B and reduced the levels of CDK2 and CCNE in neuroblastoma cells, arresting the cells at G0/G1 phase [100]. Many indirubin derivatives have been studied for their potential antisolid tumor activity [101]. Despite years of research and attempts directed at inhibiting cell cycle kinases or cell cycle regulating transcription factors, most of these approaches have not been successfully translated to the clinic as cancer therapeutics [102].

However, the inhibition of CDKs is particularly attractive from the standpoint of cancer given their key role in the cell cycle. High throughput screening and drug design based on the structure have produced several new compounds that inhibit the activity of CDKs in very specific manner. Cyclin-dependent kinases (CDKs) control cell cycle progression, RNA transcription, and apoptosis, making them interesting targets for anticancer drug development. A promising pyrazolo[1, 5-a] pyrimidine compound, devoid of ABC transporter interaction, has been identified as a highly suitable drug for further preclinical and clinical evaluation in cancer treatment [103]. Tanshinone IIA (Tan-IIA) is one of the major lipophilic components isolated from the root of Salviae Miltiorrhizae radix. Chiu et al. [104] have explored the mechanisms of cell death induced by Tan-IIA treatment in prostate cancer cells in vitro and in vivo. The G0/G1 phase arrest correlated with increase of CDK inhibitors (CDKN2A, CDKN1A, and CDKN1B) and decrease of the checkpoint proteins [104]. The effects of euphol, a tetracyclic triterpene alcohol isolated from the sap of *Euphorbia tirucalli,* were examined in T47D human breast cancer cells. Treatment of the cells with euphol resulted in decreased cell viability, which was accompanied by an accumulation of cells in the G1 phase. Euphol treatment downregulated *CCND1* expression and the hypophosphorylation of Rb. Furthermore, this effect was correlated with the downregulation of *CDK2* expression and the upregulation of the CDKIs (CDKN1A and CDKN1B), as well as reduced expression levels of CCNA and CCNB1 [105]. Treatment options for hepatocellular carcinoma (HCC) using chemotherapeutics at intermediate and advanced stages of disease are limited as frequently HCC escape from therapy and patients succumb to disease progression. The effectiveness of the novel compounds BA-12 and BP-14 that antagonize CDK1/2/5/7 and CDK9 has been studied in HCC patients, since CDKs aberrant activation is frequently observed in such patients. Inhibition of those CDKs in human HCC cell lines reduced the clonogenicity, arresting the cells in S/G2 and G2/M boundaries and inducing their apoptosis. In vivo, in mouse xenograft, this treatment also inhibited tumor development [106]. On the contrary, dysregulation of CDKN1B, due to proteolysis, frequently results in tumorigenesis. Novel compounds that inhibited CDKN1B degradation have been identified. These compounds inhibit CDKN1B ubiquitination in vitro as well as its degradation in cell cultures [107]. A novel series of 2-substituted-6-biarylmethylamino-9-cyclopentylpurine derivatives has been synthesized and screened for improved CDK inhibitory activity and antiproliferative effects. One of the most potent compounds, 6b, exhibited strong cytotoxicity in the human melanoma cell line G361 that correlated with robust CDK1 and CDK2 inhibition and caspase activation [108]. Decorin, a component of extracellular matrix, has been involved in the inhibition of cell proliferation upregulating *CDKN1A *and reactivating *CDKN1C* expression in HepG2 tumor cells [109]. CDK inhibition may be particularly successful in hematologic malignancies, which are more sensitive to cell cycling inhibition and apoptosis induction. In general, the antitumor efficacy of CDK inhibitor as monotherapy is modest, and rational combinations are being explored, including those involving other targeted agents. While selective CDK4/6 inhibition might be effective against certain malignancies, broad-spectrum CDK inhibition will likely be required for most cancers [110]. A specific inhibitor of CDK4/6 has been developed recently. PD-0332991 proved to be effective in the treatment of breast cancer [111]. dFMGEN, a novel genistein derivative, is a candidate for cancer therapy, arresting the cell cycle at G1 phase with significant reduction of CDK4 and CCND1 protein levels. This reduction is caused by CDKN2B, CDKN1A, and CDKN1B level increase and the subsequent decrease of protein levels directly suppressed Rb phosphorylation and E2F1 activity [112]. Studies of drug combination showed that flavopiridol potentiated the cytotoxicity induced by the Raf inhibitor sorafenib. This potentiation correlated with enhanced apoptosis and suppression of Rb signaling [113]. Recently the research of new targets for cancer therapy focused on *long noncoding* RNAs (lncRNAs) that are involved in the regulation of critical regulators of cell cycle, as CDKs, CCNs, and CDKIs [114]. Human tumors generally exhibit altered expression of miRNAs with oncogenic or tumor-suppressive activity. miRNA-based cancer gene therapy offers the theoretical possibility of targeting gene networks that are controlled by a single, aberrantly expressed miRNA. In experimental models, the restoration of tumor-suppressive miRNA, or sequence-specific knockdown of oncogenic miRNAs by “antagomirs,” has produced promising antitumor outcomes [115, 116].

All such data clearly show the relevance of such an approach for cancer treatment and the need to identify the appropriate combination with conventional anticancer therapy.

## 5. Nonsteroidal Anti-Inflammatory Drugs as Modulators of the Cell Cycle in Cancers

Nonsteroidal anti-inflammatory drugs (NSAIDs) are primarily used as analgesics for the relief of pain and to control inflammation [117]. NSAIDs inhibit cyclooxygenase (COX) activity and the synthesis of prostaglandins, which are mediators of inflammation. Numerous epidemiological, clinical, and laboratory studies have also suggested that NSAIDs inhibit the promotion and proliferation of some tumors [118–120]. However, the antiproliferative activity of NSAIDs requires concentrations that are 100- to 1000-fold higher than the concentrations necessary to inhibit COX activity [121]. Induction of COX-2-independent apoptosis has been described in HT29 colon cancer cells. Apoptosis was induced in these cells by the inhibition of 3-phosphoinositidedependent kinase 1 (PDK1) [122]. Moreover, apoptosis and a cell cycle blockade were observed in HCT-15 colon carcinoma cells that expressed only COX-1 (and not COX-2) [123]. NO-aspirin-induced cell cycle arrest and apoptosis of pancreatic cancer cells have been shown to occur via ROS-mediated modulation of all three MAP kinase signaling pathways and their downstream effector molecules such as CDKN1A and CCND1 [124]. Celecoxib has been shown to inhibit cancer growth independently of COX-2 expression levels with a G0/G1 cell cycle arrest and decreased levels of CCNA and CCNB1 or CCND1 depending on tumor type [125, 126]. NSAIDs increased CDKN1B by inhibiting protein degradation to suppress the proliferation of human lung cancer cells [127]. Salicylates inhibit the proliferation through upregulation of the CDKN1B and CDKN1A. This was associated with a decrease in CDK2 and to a lesser extent in CDK6 activity, thus preventing hyperphosphorylation of Rb and cell cycle progression. Similar to salicylates, however, sulindac reduced the proliferation rate of HT-29 colon carcinoma cells and caused them to accumulate in the G0/G1 phase. This was associated with reduced expression and reduced catalytic activity of cyclin-dependent kinases (CDK1, CDK2, and CDK4) [128]. These data strongly suggest the need to use such molecules as complementary therapeutic drugs besides their relevant chemopreventive role.

### 5.1. Changes of the Cell Cycle Gene Expression Profile of Gastric Cancer Cells in Response to Ibuprofen: An Anticancer Model

The primary purpose of the studies performed in our laboratory was to verify the antitumor effects of ibuprofen, a commonly used NSAID, on the MKN-45 human gastric cancer-cell line [129], where an inhibitory effect of the ibuprofen on cell proliferation was observed. These results were in agreement with data obtained in vitro, using other tumor cell lines [130, 131], as well as in vivo on xenografts of MKN-45 cells [132]. Ibuprofen was used at concentrations ranging between 400 and 800 *μ*M, and the cell proliferation rate was significantly reduced in a time- and concentration-dependent manner. Previous studies showed that concentrations of ibuprofen comparable to those used in our studies targeted a wide variety of cellular processes [122, 133–135]. Moreover, they suggested the existence of other targets or COX-independent mechanisms that could be responsible for the antiproliferative effects of ibuprofen. Our data have shown that ibuprofen is involved in the cell cycle, arresting the cells in active replication at the G0/G1 phase. Ibuprofen treatment, in fact, caused MKN-45 cells to shift from the S and G2/M phases to the G0/G1 phase, resulting in a significant accumulation of cells at the resting phase, as previously reported on different cell lines [133, 136–138]. The cell cycle changes caused in MKN-45 cells by ibuprofen correlated with alterations in cell cycle regulatory genes. The subsequent microarray analysis revealed that ibuprofen treatment for 24 h affected genes belonging to “cell cycle” pathways (Table 3). At 24 h, most of the altered genes were G1/S transition genes, such as *CDC25, CDKN1A*, and *TP53*. CDC25, one of the key regulators of cell cycle transition [139], with protooncogenic properties and carefully regulated at multiple levels [140, 141], is downregulated at all time points (since the 24 h testing) in presence of all ibuprofen concentrations, including the lower 400 *μ*M dose. *CDKN1A* gene, instead, is upregulated by more than 2-fold at 24 h, followed by downregulation at 72 h. Its upregulation, tightly controlled by TP53, is necessary to block cells in the G1 phase and allow the repair of damaged DNA before their entry into the subsequent S phase. For such role *CDKN1A* represents the key effector of p53-dependent G0/G1 phase arrest in response to different stress stimuli [142]. *TP53* levels show a similar significant upregulation for the whole ibuprofen treatment period and to all tested doses, with a late upregulation likely associated with activation of apoptotic-related genes. The upregulation of TP53 signaling pathways in the first 24 h following ibuprofen treatment has been also shown by a previous study [133]. *TP53* signaling was activated early (24 h) as a result of intracellular modifications, likely associated with oxidative stress, which would justify the ROS increase, we observed. In parallel to TP53 upregulation and cell cycle G0/G1 arrest, several S-related genes are downregulated, particularly those expressing proteins and enzymes involved in DNA replication. Regulation of S-phase-critical genes is, therefore, an important component of G1 progression to S. This coordinated regulation of S-phase genes is principally controlled by the E2F family of transcription factors [143]. Treatment with ibuprofen for 48 h at all concentrations downmodulated S-phase-critical genes, including E2F1 as well as the E2F1-regulated genes, and treatment for 72 h showed a marked modulation of apoptosis-related transcripts (Table 3). Moreover, the TP53-dependent G0/G1 arrest was paralleled with an increase in the expression of the cell cycle inhibitory protein CDKN1A, whose induction is reported to be transcriptionally upregulated by a TP53-dependent mechanism. Also the *GADD45* gene was induced by activation of the TP53-dependent pathway and likely contributed to elicit growth arrest for an effective DNA repair, or alternatively to induce apoptosis. The ineffective repair of cell damage, in fact, prevalently triggered, in our model, an apoptotic program, characterized by upregulation of caspase transcripts in the latter stages of the ibuprofen treatment.

In conclusion, this study showed that the early alterations of cell cycle regulatory genes and the later induction of apoptosis are the major mechanisms that account for the antiproliferative effects of ibuprofen on the MKN-45 human gastric cancer-cell line. Treatment with ibuprofen altered the cell cycle phase distribution by inhibiting the G1/S transition. The G0/G1 arrest was associated with a decrease in the expression of cyclins and cyclin-dependent kinases and an increase of TP53 and CDKN1A, along with downregulation of transcripts encoding enzymes involved in DNA precursor synthesis and the DNA replication system. The prolonged ibuprofen treatment and the ineffective repair of damaged cells resulted in the late upregulation of caspase transcripts with the consequent activation of an apoptotic program.

Despite the high doses of ibuprofen required to elicit the apoptotic effects reported in our studies, the implicated molecular mechanisms suggest that NSAIDs, such as ibuprofen, may be of benefit in the treatment of cancers, particularly as local treatment.

## 6. Conclusions

Several molecules and their pathways involved in the regulation of the cell cycle are currently considered as promising prognostic biomarkers and target for cancer treatment. This has been made possible by recent advances in molecular genomic and proteomic technologies. Characterization of the cell cycle pathways present in specific cancers will contribute to improving diagnosis and cancer staging, prognostic evaluation of cancer patients, and optimal combinational approaches for innovative, personalized treatment strategies.

## Figures and Tables

**Table 1:** Alterations of regulators of cell cycle, cyclins, cyclin-dependent kinases, and cyclins-dependent kinases inhibitors, prevalent in human cancers.

Regulators of cell cycle	Cycle phase or activity	Tumors
CCND1	G1	Lymphoma (90%), breast (50%) and lung carcinoma (15%), sarcoma (30%), hepatocellular carcinoma (20%), urothelial carcinoma (14%), cervical carcinoma (24%)
CCND2	G1	CLL, colorectal carcinoma, lung carcinoma (20%)
CCND3	Late G1, early S	Lymphoma (50%), retinoblastoma, urothelial carcinoma (11%)
CCNE	G1/S	Gastric and colorectal carcinoma, breast carcinoma, pancreatic carcinoma, bladder carcinoma (50%)
CCNB1	G2/M	Colorectal carcinoma, breast carcinoma, thyroid carcinoma (19%)
CCNA	S/G2	Breast carcinoma, hepatocellular carcinoma
CDK2	G1/S	Colorectal carcinoma
CDK4	G1/S	Melanoma, colorectal carcinoma, breast carcinoma, oral squamous carcinoma (50%), cervical carcinoma (26%)
CDK6	G1/S	Glioma, melanoma, oral squamous carcinoma (40%)
CDKN2B	Inhibition of CDK4/CDK6	ALL (35%), lung carcinoma (35%), melanoma, urothelial carcinoma (23%)
CDKN2A	Inhibition of CDK4/CDK6	Melanoma, glioma, breast carcinoma, nasopharyngeal carcinoma, urothelial carcinoma (23%)
CDKN1A	Inhibition of all CDKs	Melanoma, leukemia, colorectal carcinoma
CDKN1B	Inhibition of all CDKs	Melanoma, breast carcinoma, colon carcinoma

**Table 2:** Cell cycle modulators and their molecular target optimal therapeutic inhibitors.

Agent	Target
Staurosporine	CDK1, CDK2, CDK4
Flavopiridol	CDK1, CDK2, CDK4, CCND
Butyrolactone	CDK1, CDK2
Paullones	CDK1, CDK2, CDK5
Indirubin	CDK1, CDK2, CDK4, CDK5
Rapamycin	CCND, CCNA
Olomoucine	CDK1, CDK2, CDK5
Isopentenyladenine	CDK1, CDK2

**Table 3:** Functional grouping of genes up- and downregulated after ibuprofen treatment.

Symbol	Access no.	Fold change									Description
		24 h			48 h			72 h			
		400 µM	600 µM	800 µM	400 µM	600 µM	800 µM	400 µM	600 µM	800 µM	
Cell cycle checkpoint											
G1/S DNA damage checkpoint											
CDC25A	NM_001789	—	—	−2,090	—	—	—	−2,408	—	—	Cell division cycle 25 homolog A (*S. pombe*)
CDC25B	NM_021873	—	—	—	—	—	—	−2,980	−2,686	−2,415	Cell division cycle 25 homolog B (*S. pombe*)
CDC25C	NM_001790	—	—	−2,155	−2,306	—	−2,050	−3,588	—	—	Cell division cycle 25 homolog C (*S. pombe*)
p53-dependent G1/S DNA Damage checkpoint											
TP53	NM_000546	—	2,447	2,503	—	—	—	3,150	—	3,650	Tumor protein p53
CDKN1A	NM_078467	—	3,074	2,789	—	—	—	—	−2,254	−2,203	Cyclin-dependent kinase inhibitor 1A (p21, Cip1)
GADD45A	NM_001924	—	—	—	—	—	—	4,194	2,493	2,047	Growth arrest and DNA-damage-inducible, alpha
GADD45B	NM_015675	2,352	—	2,191	2,864	2,792	2,899	4,016	5,479	5,200	Growth arrest and DNA-damage-inducible, beta
GADD45G	NM_006705	—	2,208	2,661		2,379	4,796	5,081	14,121	22,532	Growth arrest and DNA-damage-inducible, gamma
TP53INP1	NM_033285	2,033	3,475	5,494	3,071	—	—	—	—	—	Tumor protein 53 inducible nuclear protein 1
TP53I3	NM_004881	—	—	—	—	—	2,214	—	—	2,406	Tumor protein p53 inducible protein 3
MDM2	NM_002392	2,534	—	2,868	3,019	4,111	3,165	—	4,369	—	Mdm2 p53 binding protein homolog (mouse)
Cell cycle mitotic											
G1/S transition											
CCNE1	NM_001238	—	—	—	—	−2,328	−2,652	−3,523	—	—	Cyclin E1
CDKN1A	NM_078467	—	3,074	2,789	—	—	—	—	−2,254	−2,203	Cyclin-dependent kinase inhibitor 1A (p21, Cip1)
CDKN1B	NM_004064	—	—	—	—	—	2,477	2,432	3,367	4,938	Cyclin-dependent kinase inhibitor 1B (p27, Kip1)
CDC2	NM_001786	—	—	−2,540	−3,260	−2,250	−3,940	−5,820	−2,730	−3,420	Cell division cell cycle 2
RB1	NM_000321	—	—	—	—	—	—	—	2,872	2,473	Retinoblastoma 1
DNA replication / E2F mediated regulation of DNA replication											
E2F1	NM_005225	—	—	—	−2,283	−3,073	−2,740	−7,552	−4,090	−3,215	E2F transcription factor 1
CDT1	NM_030928	—	—	—	−2,571	−2,727	−2,332	−5,931	−3,269	−2,202	Chromatin licensing and DNA replication factor 1
PCNA	NM_002592	—	—	—	−2,073	−2,320	−3,481	−3,900	−5,797	−8,575	Proliferating cell nuclear antigen
DHFR	NM_000791	—	—	—	—	−2,466	−2,157	−2,376	−4,963	−7,712	Dihydrofolate reductase
TYMS	NM_001071	—	—	—	−2,088	−3,595	−4,872	−8,869	−9,976	−11,282	Thymidylate synthase
POLA2	NM_002689	—	—	—	—	—	—	−3,763	−4,911	−6,198	DNA polymerase alpha2
POLA1	NM_016937	—	—	—	—	—	—	−2,098	—	−2,014	DNA polymerase alpha 1
POLD2	NM_006230	—	—	—	—	−2,794	−2,403	−2,611	−3,001	−2,114	DNA polymerase delta 2
POLD3	NM_006591	—	—	−2,244	—	−2,135	—	−2,992	—	—	DNA polymerase delta 3
POLE2	NM_002692	—	—	−2,033	−3,289	−6,780	−6,793	−8,488	−10,860	−11,678	DNA polymerase epsilon 2
POLE4	NM_019896	—	—	—	—	—	−2,154	—	−2,118	—	DNA polymerase epsilon 4
ORC1L	NM_004153	—	—	—	−2,914	−2,588	−2,538	−5,605	−2,337	—	Origin recognition complex, subunit 1-like (yeast)
RRM2	NM_001034	—	—	—	−2,467	−2,485	−4,266	−6,902	−4,767	−4,299	Ribonucleotide reductase M2 subunit
Regulation of DNA replication											
ORC2L	NM_006190	—	—	—	—	—	—	2,288	2,727	2,570	Origin recognition complex, subunit 2-like (yeast)
ORC4L	NM_002552	—	—	—	—	—	3,216	2,171	—	—	Origin recognition complex, subunit 4-like (yeast)
ORC5L	NM_181747	—	—	—	—	—	—	2,209	2,902	2,765	Origin recognition complex, subunit 5-like (yeast)
ORC6L	NM_014321	—	—	—	−2,348	—	—	—	−2,415	—	Origin recognition complex, subunit 6-like (yeast)
MCM2	NM_004526	—	—	—	−2,664	−3,036	−2,943	−6,176	−4,620	−3,494	Minichromosome maintenance complex component 2
MCM3	NM_002388	—	—	—	−2,105	−3,104	−3,458	−4,038	−3,948	−4,356	Minichromosome maintenance complex component 3
MCM4	NM_005914	—	—	—	−2,267	−2,266	−2,464	−3,923	−5,040	−7,344	Minichromosome maintenance complex component 4
MCM5	NM_006739	—	—	—	−2,360	−3,103	−3,392	−7,464	−4,613	−3,677	Minichromosome maintenance complex component 5
MCM6	NM_005915	—	—	—	−2,320	−2,056	—	—	—	—	Minichromosome maintenance complex component 6
MCM7	NM_182776	—	—	−2,175	−2,563	—	—	−2,617	−2,024	−2,345	Minichromosome maintenance complex component 7
TK1	NM_003258	—	—	−2,384	−3,839	−5,606	−7,557	−24,478	−7,567	−5,991	Thymidine kinase 1, soluble
FEN1	NM_004111	—	—	−2,008	−2,268	—	−2,110	−4,470	−3,294	−2,587	Flap structure-specific endonuclease 1
LIG1	NM_000234	—	—	—	−2,626	−3,753	−3,867	−8,964	−6,958	−5,640	DNA Ligase 1
Apoptosis											
CASP3	NM_004346	—	—	—	—	—	2,097	—	2,239	2,112	Caspase-3
CASP7	NM_033339	—	—	—	—	2,124	2,097	2,050	2,335	2,393	Caspase-7
CASP8	NM_033356	—	—	—	—	—	—	3,686	2,420	3,056	Caspase-8
CASP9	NM_001229	—	—	—	—	—	—	—	2,221	2,257	Caspase-9
CASP10	NM_032977	—	—	—	—	—	—	—	3,617	4,514	Caspase-10
DFFA	NM_213566	—	—	—	—	—	—	—	—	2,034	DNA fragmentation factor, 45kDa, alpha polypeptide
DFFB	NM_001004285	—	—	—	—	—	—	4,015	2,121	4,583	DNA fragmentation factor, 40 kD, beta polypeptide (caspase-activated DNase)

## References

[1] Sherr CJ (1996). Cancer cell cycles. *Science*.

[2] Hartwell LH, Kastan MB (1994). Cell cycle control and cancer. *Science*.

[3] Dictor M, Ehinger M, Mertens F, Åkervall J, Wennerberg J (1999). Abnormal cell cycle regulation in malignancy. *American Journal of Clinical Pathology*.

[4] van den Heuvel S, Harlow E (1993). Distinct roles for cyclin-dependent kinases in cell cycle control. *Science*.

[5] Weinberg RA (1995). The retinoblastoma protein and cell cycle control. *Cell*.

[6] Draetta G (1990). Cell cycle control in eukaryotes molecular mechanisms of cdc2 activation. *Trends in Biochemical Sciences*.

[7] Sherr CJ, Roberts JM (1999). CDK inhibitors: positive and negative regulators of G1-phase progression. *Genes and Development*.

[8] Sausville EA, Johnson J, Alley M, Zaharevitz D, Senderowicz AM (2000). Inhibition of CDKs as a therapeutic modality. *Annals of the New York Academy of Sciences*.

[9] Morgan DO (1995). Principles of CDK regulation. *Nature*.

[10] Xiong Y, Hannon GJ, Zhang H, Casso D, Kobayashi R, Beach D (1993). p21 is a universal inhibitor of cyclin kinases. *Nature*.

[11] Dyson N (1998). The regulation of E2F by pRB-family proteins. *Genes and Development*.

[12] Hatakeyama M, Weinberg RA (1995). The role of RB in cell cycle control. *Progress in Cell Cycle Research*.

[13] Sherr CJ (1994). G1 phase progression: cycling on cue. *Cell*.

[14] Nilsson I, Hoffmann I (2000). Cell cycle regulation by the Cdc25 phosphatase family. *Progress in Cell Cycle Research*.

[15] Hartwell LH, Weinert TA (1989). Checkpoints: controls that ensure the order of cell cycle events. *Science*.

[16] Buolamwini JK (2000). Cell cycle molecular targets in novel anticancer drug discovery. *Current Pharmaceutical Design*.

[17] Hunter T, Pines J (1994). Cyclins and cancer II: cyclin D and CDK inhibitors come of age. *Cell*.

[18] Karp JE, Broder S (1995). Molecular foundations of cancer: new targets for intervention. *Nature Medicine*.

[19] Draetta GF (1994). Mammalian G1 cyclins. *Current Opinion in Cell Biology*.

[20] Dreyling MH, Bullinger L, Ott G (1997). Alterations of the cyclin D1/p16-pRB pathway in mantle cell lymphoma. *Cancer Research*.

[21] Hall M, Peters G (1996). Genetic alterations of cyclins, cyclin-dependent kinases, and Cdk inhibitors in human cancer. *Advances in Cancer Research*.

[22] Leach FS, Elledge SJ, Sherr CJ (1993). Amplification of cyclin genes in colorectal carcinomas. *Cancer Research*.

[23] Motokura T, Arnold A (1993). Cyclin D and oncogenesis. *Current Opinion in Genetics and Development*.

[24] Davis MA, Stürzl M, Blasig C (1997). Expression of human herpesvirus 8-encoded cyclin D in Kaposi’s sarcoma spindle cells. *Journal of the National Cancer Institute*.

[25] Niklaus AL, Aubuchon M, Zapantis G (2007). Assessment of the proliferative status of epithelial cell types in the endometrium of young and menopausal transition women. *Human Reproduction*.

[26] Keyomarsi K, Herliczek TW (1997). The role of cyclin E in cell proliferation, development and cancer. *Progress in Cell Cycle Research*.

[27] Gudas JM, Payton M, Thukral S (1999). Cyclin E2, a novel G1 cyclin that binds Cdk2 and is aberrantly expressed in human cancers. *Molecular and Cellular Biology*.

[28] Buckley MF, Sweeney KJE, Hamilton JA (1993). Expression and amplification of cyclin genes in human breast cancer. *Oncogene*.

[29] Wang J, Zindy F, Chenivesse X, Lamas E, Henglein B, Brechot C (1992). Modification of cyclin A expression by hepatitis B virus DNA integration in a hepatocellular carcinoma. *Oncogene*.

[30] Kim JH, Kang MJ, Park CU (1999). Amplified CDK2 and cdc2 activities in primary colorectal carcinoma. *Cancer*.

[31] Ikeda K, Monden T, Tsujie M (1996). Cyclin D, CDK4 and p16 expression in colorectal cancer. *Nippon Rinsho*.

[32] Costello JF, Plass C, Arap W (1997). Cyclin-dependent kinase 6 (CDK6) amplification in human gliomas identified using two-dimensional separation of genomic DNA. *Cancer Research*.

[33] Kamb A, Gruis NA, Weaver-Feldhaus J (1994). A cell cycle regulator potentially involved in genesis of many tumor types. *Science*.

[34] Hayashi N, Sugimoto Y, Tsuchiya E, Ogawa M, Nakamura Y (1994). Somatic mutations of the MTS (Multiple Tumor Suppressor) 1/CDK4I (Cyclin-Dependent Kinase-4 Inhibitor) gene in human primary non-small cell lung carcinomas. *Biochemical and Biophysical Research Communications*.

[35] Cairns P, Mao L, Merlo A (1994). Rates of p16 (MTS1) mutations in primary tumors with 9p loss. *Science*.

[36] Drexler HG (1998). Review of alterations of the cyclin-dependent kinase inhibitor INK4 family genes p15, p16, p18 and p19 in human leukemia-lymphoma cells. *Leukemia*.

[37] Meltzer SJ (1996). The molecular biology of esophageal carcinoma. *Recent Results in Cancer Research*.

[38] Takamura H, Fushida S, Hashimoto T, Yagi M, Miyazaki I (1996). Analysis of the p16INK4, p15INK4B genes abnormality and the amplification of cyclin D1 gene in esophageal cancer. *Nippon Rinsho*.

[39] Cordon-Cardo C (1995). Mutation of cell cycle regulators: biological and clinical implications for human neoplasia. *American Journal of Pathology*.

[40] Loda M, Cukor B, Tam SW (1997). Increased proteasome-dependent degradation of the cyclin-dependent kinase inhibitor p27 in aggressive colorectal carcinomas. *Nature Medicine*.

[41] Barbieri F, Cagnoli M, Ragni N, Pedullà F, Foglia G, Alama A (1997). Expression of cyclin D1 correlates with malignancy in human ovarian tumours. *British Journal of Cancer*.

[42] Zhang T, Nanney LB, Luongo C (1997). Concurrent overexpression of cyclin D1 and cyclin-dependent kinase 4 (Cdk4) in intestinal adenomas from multiple intestinal neoplasia (min) mice and human familial adenomatous polyposis patients. *Cancer Research*.

[43] Nakagawa K, Conrad NK, Williams JP, Johnson BE, Kelley MJ (1995). Mechanism of inactivation of CDKN2 and MTS2 in non-small cell lung cancer and association with advanced stage. *Oncogene*.

[44] Bartkova J, Lukas J, Guldberg P (1996). The p16-cyclin D/Cdk4-pRb pathway as a functional unit frequently altered in melanoma pathogenesis. *Cancer Research*.

[45] Honoki K, Fujii H, Tohma Y (2012). Comparison of gene expression profiling in sarcomas and mesenchymal stem cells identifies tumorigenic pathways in chemically induced rat sarcoma model. *Oncology*.

[46] Yamamoto H, Monden T, Miyoshi H (1998). Cdk2/cdc2 expression in colon carcinogenesis and effects of cdk2/cdc2 inhibitor in colon cancer cells. *International Journal of Oncology*.

[47] Chiarle R, Voena C, Ambrogio C, Piva R, Inghirami G (2008). The anaplastic lymphoma kinase in the pathogenesis of cancer. *Nature Reviews Cancer*.

[48] Marone M, Scambia G, Giannitelli C (1998). Analysis of cyclin E and CDK2 in ovarian cancer: gene amplification and RNA overexpression. *International Journal of Cancer*.

[49] Easton J, Wei T, Lahti JM, Kidd VJ (1998). Disruption of the cyclin D/cyclin-dependent kinase/INK4/retinoblastoma protein regulatory pathway in human neuroblastoma. *Cancer Research*.

[50] Zuo L, Weger J, Yang Q (1996). Germline mutations in the p16(INK4a) binding domain of CDK4 in familial melanoma. *Nature Genetics*.

[51] Obaya AJ, Sedivy JM (2002). Regulation of cyclin-Cdk activity in mammalian cells. *Cellular and Molecular Life Sciences*.

[52] Matsuda Y (2008). Molecular mechanism underlying the functional loss of cyclindependent kinase inhibitors p16 and p27 in hepatocellular carcinoma. *World Journal of Gastroenterology*.

[53] Lee RJ, Albanese C, Stenger RJ (1999). pp60(v-src) induction of cyclin D1 requires collaborative interactions between the extracellular signal-regulated kinase, p38, and Jun kinase pathways: a role for cAMP response element-binding protein and activating transcription factor-2 in pp60(v-src) signaling in breast cancer cells. *The Journal of Biological Chemistry*.

[54] Albanese C, Johnson J, Watanabe G (1995). Transforming p21(ras) mutants and c-Ets-2 activate the cyclin D1 promoter through distinguishable regions. *The Journal of Biological Chemistry*.

[55] Lovec H, Grzeschiczek A, Kowalski M, Möröy T (1994). Cyclin D1/bcl-1 cooperates with myc genes in the generation of B-cell lymphoma in transgenic mice. *The EMBO Journal*.

[56] Daksis JI, Lu RY, Facchini LM, Marhin WW, Penn LJZ (1994). Myc induces cyclin D1 expression in the absence of de novo protein synthesis and links mitogen-stimulated signal transduction to the cell cycle. *Oncogene*.

[57] de Boer CJ, van Krieken JHJM, Schuuring E, Kluin PM (1997). Bcl-1/cyclin D1 in malignant lymphoma. *Annals of Oncology*.

[58] Fiancette R, Amin R, Truffinet V (2010). A myeloma translocation-like model associating CCND1 with the immunoglobulin heavy-chain locus 3′ enhancers does not promote by itself B-cell malignancies. *Leukemia Research*.

[59] Wang TC, Cardiff RD, Zukerberg L, Lees E, Arnold A, Schmidt EV (1994). Mammary hyperplasia and carcinoma in MMTV-cyclin D1 transgenic mice. *Nature*.

[60] Arnold A, Papanikolaou A (2005). Cyclin D1 in breast cancer pathogenesis. *Journal of Clinical Oncology*.

[61] Bodrug SE, Warner BJ, Bath ML, Lindeman GJ, Harris AW, Adams JM (1994). Cyclin D1 transgene impedes lymphocyte maturation and collaborates in lymphomagenesis with the myc gene. *The EMBO Journal*.

[62] Möröy T, Geisen C (2004). Cyclin E. *International Journal of Biochemistry and Cell Biology*.

[63] Keyomarsi K, Conte D, Toyofuku W, Fox MP (1995). Deregulation of cyclin E in breast cancer. *Oncogene*.

[64] Schraml P, Bucher C, Bissig H (2003). Cyclin E overexpression and amplification in human tumours. *The Journal of Pathology*.

[65] Lida H, Towatari M, Tanimoto M, Morishita Y, Kodera Y, Saito H (1997). Overexpression of cyclin E in acute myelogenous leukemia. *Blood*.

[66] Michalides R, van Veelen N, Hart A, Loftus B, Wientjens E, Balm A (1995). Overexpression of cyclin D1 correlates with recurrence in a group of forty-seven operable squamous cell carcinomas of the head and neck. *Cancer Research*.

[67] Noel EE, Yeste-Velasco M, Mao X (2010). The association of CCND1 overexpression and cisplatin resistance in testicular germ cell tumors and other cancers. *American Journal of Pathology*.

[68] Gansauge S, Gansauge F, Ramadani M (1997). Overexpression of cyclin D1 in human pancreatic carcinoma is associated with poor prognosis. *Cancer Research*.

[69] Mineta H, Miura K, Takebayashi S (2000). Cyclin D1 overexpression correlates with poor prognosis in patients with tongue squamous cell carcinoma. *Oral Oncology*.

[70] Sutter T, Doi S, Carnevale KA, Arber N, Weinstein IB (1997). Expression of cyclins D1 and E in human colon adenocarcinomas. *Journal of Medicine*.

[71] Tzankov A, Gschwendtner A, Augustin F (2006). Diffuse large B-cell lymphoma with overexpression of cyclin E substantiates poor standard treatment response and inferior outcome. *Clinical Cancer Research*.

[72] Wang L, Shao Z (2006). Cyclin E expression and prognosis in breast cancer patients: a meta-analysis of published studies. *Cancer Investigation*.

[73] Zhou YJ, Xie YT, Gu J, Yan L, Guan GX, Liu X (2011). Overexpression of cyclin e isoforms correlates with poor prognosis in rectal cancer. *European Journal of Surgical Oncology*.

[74] Bortner DM, Rosenberg MP (1997). Induction of mammary gland hyperplasia and carcinomas in transgenic mice expressing human cyclin E. *Molecular and Cellular Biology*.

[75] Porter PL, Malone KE, Heagerty PJ (1997). Expression of cell-cycle regulators p27(Kip1) and cyclin E, alone and in combination, correlate with survival in young breast cancer patients. *Nature Medicine*.

[76] Akama Y, Yasui W, Yokozaki H (1995). Frequent amplification of the cyclin E gene in human gastric carcinomas. *Japanese Journal of Cancer Research*.

[77] Kim HK, Park I, Heo DS (2001). Cyclin E overexpression as an independent risk factor of visceral relapse in breast cancer. *European Journal of Surgical Oncology*.

[78] Tsihlias J, Kapusta L, Slingerland J (1999). The prognostic significance of altered cyclin-dependent kinase inhibitors in human cancer. *Annual Review of Medicine*.

[79] Straume O, Sviland L, Akslen LA (2000). Loss of nuclear p16 protein expression correlates with increased tumor cell proliferation (Ki-67) and poor prognosis in patients with vertical growth phase melanoma. *Clinical Cancer Research*.

[80] Zhang H, Sun XF (2001). Loss of p27 expression predicts poor prognosis in patients with Dukes’ B stage or proximal colorectal cancer. *International Journal of Oncology*.

[81] Juuti A, Nordling S, Louhimo J, Lundin J, Von Boguslawski K, Haglund C (2003). Loss of p27 expression is associated with poor prognosis in stage I-II pancreatic cancer. *Oncology*.

[82] Tenjo T, Toyoda M, Okuda J (2000). Prognostic significance of p27(kip1) protein expression and spontaneous apoptosis in patients with colorectal adenocarcinomas. *Oncology*.

[83] Hu Y, Liao M, Ding J, Zhou J, Xu K (2002). Co-deletion of both p15/p16 genes correlates with poor prognosis non-small cell lung cnacer. *Chinese Journal of Cancer Research*.

[84] Matsuda Y, Ichida T, Genda T, Yamagiwa S, Aoyagi Y, Asakura H (2003). Loss of p16 contributes to p27 sequestration by cyclin D l-cyclin-dependent kinase 4 complexes and poor prognosis in hepatocellular carcinoma. *Clinical Cancer Research*.

[85] Fujiwara S, Noguchi T, Takeno S, Kimura Y, Fumoto S, Kawahara K (2008). Hypermethylation of p16 gene promoter correlates with loss of p16 expression that results in poorer prognosis in esophageal squamous cell carcinomas. *Diseases of the Esophagus*.

[86] Kees UR, Burton PR, Lü C, Baker DL (1997). Homozygous deletion of the p16/MTS1 gene in pediatric acute lymphoblastic leukemia is associated with unfavorable clinical outcome. *Blood*.

[87] Yamada Y, Hatta Y, Murata K (1997). Deletions of p15 and/or p16 genes as a poor-prognosis factor in adult T-cell leukemia. *Journal of Clinical Oncology*.

[88] Tien H, Tang J, Tsay W (2001). Methylation of the p15INK4B gene in myelodysplastic syndrome: it can be detected early at diagnosis or during disease progression and is highly associated with leukaemic transformation. *British Journal of Haematology*.

[89] Tsihlias J, Kapusta L, Slingerland J (1999). The prognostic significance of altered cyclin-dependent kinase inhibitors in human cancer. *Annual Review of Medicine*.

[90] Wander SA, Zhao D, Slingerland JM (2011). p27: a barometer of signaling deregulation and potential predictor of response to targeted therapies. *Clinical Cancer Research*.

[91] Takano Y, Kato Y, van Diest PJ, Masuda M, Mitomi H, Okayasu I (2000). Cyclin D2 overexpression and lack of p27 correlate positively and cyclin E inversely with a poor prognosis in gastric cancer cases. *American Journal of Pathology*.

[92] Pertia A, Nikoleishvili D, Trsintsadze O, Gogokhia N, Managadze L, Chkhotua A (2007). Loss of p27(Kip1) CDKI is a predictor of poor recurrence-free and cancer-specific survival in patients with renal cancer. *International Urology and Nephrology*.

[93] Abbas T, Dutta A (2009). P21 in cancer: intricate networks and multiple activities. *Nature Reviews Cancer*.

[94] Giglio S, Cirombella R, Amodeo R (2013). MicroRNA miR-24 promotes cell proliferation by targeting the CDKs inhibitors p27Kip1 and p16INK4a. *Journal of Cellular Physiology*.

[95] Ivanovska I, Ball AS, Diaz RL (2008). MicroRNAs in the miR-106b family regulate p21/CDKN1A and promote cell cycle progression. *Molecular and Cellular Biology*.

[96] Petrocca F, Visone R, Onelli MR (2008). E2F1-regulated microRNAs impair TGFbeta-dependent cell-cycle arrest and apoptosis in gastric cancer. *Cancer Cell*.

[97] Wu S, Huang S, Ding J (2010). Multiple microRNAs modulate p21Cip1/Waf1 expression by directly targeting its 3′ untranslated region. *Oncogene*.

[98] Koh J, Kubota T, Koyama T (2002). Combined antitumor activity of 7-hydroxystaurosporine (UCN-01) and tamoxifen against human breast carcinoma in vitro and in vivo. *Breast Cancer*.

[99] Kasuya Y, Hosaka Y, Matsushima H, Goto T, Kitamura T, Okuyama A (2003). Prominent induction of cyclin B1 in G2/M renal cancer cells with butyrolactone 1. *International Journal of Urology*.

[100] Liao XM, Leung KN (2013). Indirubin-3′-oxime induces mitochondrial dysfunction and triggers growth inhibition and cell cycle arrest in human neuroblastoma cells. *Oncology Reports*.

[101] Kim S, Choi SJ, Kim Y, Kuh H (2009). Anti-tumor activity of noble indirubin derivatives in human solid tumor models in vitro. *Archives of Pharmacal Research*.

[102] Lapenna S, Giordano A (2009). Cell cycle kinases as therapeutic targets for cancer. *Nature Reviews Drug Discovery*.

[103] Kaliszczak M, Patel H, Kroll SH (2013). Development of a cyclin-dependent kinase inhibitor devoid of ABC transporter-dependent drug resistance. *British Journal of Cancer*.

[104] Chiu SC, Huang SY, Chen SP (2013). Tanshinone IIA inhibits human prostate cancer cells growth by induction of endoplasmic reticulum stress in vitro and in vivo. *Prostate Cancer and Prostatic Diseases*.

[105] Wang L, Wang G, Yang D (2013). Euphol arrests breast cancer cells at the G1 phase through the modulation of cyclin D1, p21 and p27 expression. *Molecular Medicine Reports*.

[106] Haider C, Grubinger M, Reznícková E (2013). Novel inhibitors of cyclin-dependent kinases combat hepatocellular carcinoma without inducing chemoresistance. *Molecular Cancer Therapeutics*.

[107] Ooi LC, Watanabe N, Futamura Y (2013). Identification of small molecule inhibitors of p27Kip1 ubiquitination by high-throughput screening. *Cancer Science*.

[108] Gurcký T, Jorda R, Zatloukal M (2013). A novel series of highly potent 2, 6, 9-trisubstituted purine cyclin-dependent kinase inhibitors. *Journal of Medicinal Chemistry*.

[109] Hamid AS, Li J, Wang Y (2013). Recombinant human decorin upregulates p57KIP2 expression in HepG2 hepatoma cell lines. *Molecular Medicine Reports*.

[110] Bose P, Simmons GL, Grant S (2013). Cyclin-dependent kinase inhibitor therapy for hematologic malignancies. *Expert Opinion on Investigational Drugs*.

[111] Dean JL, Thangavel C, McClendon AK, Reed CA, Knudsen ES (2010). Therapeutic CDK4/6 inhibition in breast cancer: key mechanisms of response and failure. *Oncogene*.

[112] Peng B, Cao J, Yi S (2013). Inhibition of proliferation and induction of G1-phase cell-cycle arrest by dFMGEN, a novel genistein derivative, in lung carcinoma A549 cells. *Drug and Chemical Toxicology*.

[113] Nagaria TS, Williams JL, Leduc C (2013). Flavopiridol synergizes with sorafenib to induce cytotoxicity and potentiate antitumorigenic activity in EGFR/HER-2 and mutant RAS/RAF breast cancer model systems. *Neoplasia*.

[114] Kitagawa M, Kitagawa K, Kotabe Y (2013). Cell cycle regulation by long non-coding RNAs. *Cellular and Molecular Life Sciences*.

[115] Tong AW, Nemunaitis J (2008). Modulation of miRNA activity in human cancer: a new paradigm for cancer gene therapy?. *Cancer Gene Therapy*.

[116] Segura MF, Greenwald HS, Hanniford D (2012). MicroRNA and cutaneous melanoma: from discovery to prognosis and therapy. *Carcinogenesis*.

[117] Hinz B, Brune K (2007). Antipyretic analgesics: nonsteroidal antiinflammatory drugs, selective COX-2 inhibitors, paracetamol and pyrazolinones. *Handbook of Experimental Pharmacology*.

[118] Kokoska ER, Smith GS, Wolff AB, Deshpande Y, Miller TA (1999). Nonsteroidal anti-inflammatory drugs attenuate epidermal growth factor- induced proliferation independent of prostaglandin synthesis inhibition. *Journal of Surgical Research*.

[119] Giardiello FM (1996). NSAID-induced polyp regression in familial adenomatous polyposis patients. *Gastroenterology Clinics of North America*.

[120] Barnes CJ, Cameron IL, Hardman WE, Lee M (1998). Non-steroidol anti-inflammatory drug effect on crypt cell proliferation and apoptosis during initiation of rat colon carcinogenesis. *British Journal of Cancer*.

[121] Giovannucci E, Egan KM, Hunter DJ (1995). Aspirin and the risk of colorectal cancer in women. *The New England Journal of Medicine*.

[122] de Groot DJA, de Vries EGE, Groen HJM, de Jong S (2007). Non-steroidal anti-inflammatory drugs to potentiate chemotherapy effects: from lab to clinic. *Critical Reviews in Oncology/Hematology*.

[123] Janssen A, Maier TJ, Schiffmann S (2006). Evidence of COX-2 independent induction of apoptosis and cell cycle block in human colon carcinoma cells after S- or R-ibuprofen treatment. *European Journal of Pharmacology*.

[124] Zhou H, Huang L, Sun Y, Rigas B (2009). Nitric oxide-donating aspirin inhibits the growth of pancreatic cancer cells through redox-dependent signaling. *Cancer Letters*.

[125] Maier TJ, Schilling K, Schmidt R, Geisslinger G, Grösch S (2004). Cyclooxygenase-2 (COX-2)-dependent and -independent anticarcinogenic effects of celecoxib in human colon carcinoma cells. *Biochemical Pharmacology*.

[126] Narayanan BA, Condon MS, Bosland MC, Narayanan NK, Reddy BS (2003). Suppression of N-methyl-N-nitrosourea/testosterone-induced rat prostate cancer growth by celecoxib: effects on cyclooxygenase-2, cell cycle regulation, and apoptosis mechanism(s). *Clinical Cancer Research*.

[127] Huang Y, Chuang L, Hung W (2002). Mechanisms underlying nonsteroidal anti-inflammatory drug-induced p27Kip1 expression. *Molecular Pharmacology*.

[128] Shiff SJ, Qiao L, Tsai L-L, Rigas B (1995). Sulindac sulfide, an aspirin-like compound, inhibits proliferation, causes cell cycle quiescence, and induces apoptosis in HT-29 colon adenocarcinoma cells. *Journal of Clinical Investigation*.

[129] Bonelli P, Tuccillo FM, Calemma R (2011). Changes in the gene expression profile of gastric cancer cells in response to ibuprofen: a gene pathway analysis. *Pharmacogenomics Journal*.

[130] Takada Y, Bhardwaj A, Potdar P, Aggarwal BB (2004). Nonsteroidal anti-inflammatory agents differ in their ability to suppress NF-*κ*B activation, inhibition of expression of cyclooxygenase-2 and cyclin D1, and abrogation of tumor cell proliferation. *Oncogene*.

[131] Thurnher D, Bakroeva M, Formanek M, Knerer B, Kornfehl J (2001). Non-steroidal anti-inflammatory drugs inhibit telomerase activity in head and neck squamous carcinoma cell lines. *Head and Neck*.

[132] Sawaoka H, Kawano S, Tsuji S (1998). Cyclooxygenase-2 inhibitors suppress the growth of gastric cancer xenografts via induction of apoptosis in nude mice. *American Journal of Physiology—Gastrointestinal and Liver Physiology*.

[133] Aryankalayil MJ, Palayoor ST, Cerna D, Falduto MT, Magnuson SR, Coleman CN (2009). NS-398, ibuprofen, and cyclooxygenase-2 RNA interference produce significantly different gene expression profiles in prostate cancer cells. *Molecular Cancer Therapeutics*.

[134] Redpath M, Marques CMG, Dibden C, Waddon A, Lalla R, MacNeil S (2009). Ibuprofen and hydrogel-released ibuprofen in the reduction of inflammation-induced migration in melanoma cells. *British Journal of Dermatology*.

[135] Quann EJ, Khwaja F, Djakiew D (2007). The p38 MAPK pathway mediates aryl propionic acid-induced messenger RNA stability of p75NTR in prostate cancer cells. *Cancer Research*.

[136] Andrews J, Djakiew D, Krygier S, Andrews P (2002). Superior effectiveness of ibuprofen compared with other NSAIDs for reducing the survival of human prostate cancer cells. *Cancer Chemotherapy and Pharmacology*.

[137] Tegeder I, Pfeilschifter J, Geisslinger G (2001). Cyclooxygenase-independent actions of cyclooxygenase inhibitors. *The FASEB Journal*.

[138] Masferrer JL, Leahy KM, Koki AT (2000). Antiangiogenic and antitumor activities of cyclooxygenase-2 inhibitors. *Cancer Research*.

[139] Ekholm SV, Reed SI (2000). Regulation of G1 cyclin-dependent kinases in the mammalian cell cycle. *Current Opinion in Cell Biology*.

[140] Viallard JF, Lacombe F, Belloc F, Pellegrin JL, Reiffers J (2001). Molecular mechanisms controlling the cell cycle: main considerations and implications in oncology. *Cancer/Radiotherapie*.

[141] Kristjánsdóttir K, Rudolph J (2004). Cdc25 phosphatases and cancer. *Chemistry and Biology*.

[142] Waldman T, Kinzler KW, Vogelstein B (1995). p21 Is necessary for the p53-mediated G1 arrest in human cancer cells. *Cancer Research*.

[143] Cam H, Dynlacht BD (2003). Emerging roles for E2F: beyond the G1/S transition and DNA replication. *Cancer Cell*.

